# LncRNAs could play a vital role in osteosarcoma treatment: Inhibiting osteosarcoma progression and improving chemotherapy resistance

**DOI:** 10.3389/fgene.2022.1022155

**Published:** 2023-01-16

**Authors:** Shi Xiaotong, Li Xiao, Liao Shiyu, Bi Zhiguo, Feng Chunyang, Liu Jianguo

**Affiliations:** ^1^ Department of Orthopedics, The First Hospital of Jilin University, Changchun, China; ^2^ Department of Obstetrics and Gynecology, Renji Hospital of Shanghai Jiao Tong University, Shanghai, China

**Keywords:** long non-coding RNA (LncRNA), osteosarcoma-pathology, progression, chemotherapy resistance, regulatory mechanism

## Abstract

Osteosarcoma (OS) is one of the most common primary solid malignant tumors in orthopedics, and its main clinical treatments are surgery and chemotherapy. However, a wide surgical resection range, functional reconstruction of postoperative limbs, and chemotherapy resistance remain as challenges for patients and orthopedists. To address these problems, the discovery of new effective conservative treatments is important. Long non-coding RNAs (lncRNAs) are RNAs longer than 200 nucleotides in length that do not encode proteins. Researchers have recently found that long non-coding RNAs are closely associated with the development of OS, indicating their potentially vital role in new treatment methods for OS. This review presents new findings regarding the association of lncRNAs with OS and summarizes potential clinical applications of OS with lncRNAs, including the downregulation of oncogenic lncRNAs, upregulation of tumor suppressive lncRNAs, and lncRNAs-based treatment to improve chemotherapy resistance. We hope these potential methods will be translated into clinical applications and greatly reduce patient suffering.

## 1 Introduction

Osteosarcoma (OS) is the most common primary solid malignant tumor in orthopedics and is derived from primitive mesenchymal cells (Ritter and Bielack, 2010). Long diaphyses such as the distal femur, proximal tibia, and proximal humerus are most often affected by OS. Data show that 50% of OS occurs around the knee, which can seriously impair patient motor ability (Arndt and Crist, 1999; Bielack et al., 2002; Bielack et al., 2009). The annual incidence of OS is 2–3 million people, with adolescents most at risk (Corre et al., 2020). Although most patients initially present local pain and swelling, pathological fracture of the affected limbs and metastasis-related symptoms can occur early in the disease as OS is highly aggressive and metastasizes early (Bielack and CarrleCasali, 2009). In addition, the 5-year survival rate of patients with early metastasis is usually <20%, with lung metastasis the most likely to occur (Wang et al., 2020a). At present, due to rapid tumor development and the high invasion level of local tissue, surgery combined with chemotherapy is the main treatment for patients with OS (Schwarz et al., 2009; Ta et al., 2009). However, early metastasis, a wide surgical resection range, a high risk of postoperative recurrence, and difficult postoperative function reconstruction challenge OS treatment methods (Grimer, 2005; Gosheger et al., 2006; Bielack et al., 2009). Therefore, new clinical methods are needed, including accurate monitoring methods for early diagnosis and postoperative surveillance, less invasive treatment methods to avoid massive limb function loss, and more effective methods to lower postoperative recurrence.

LncRNAs are RNAs >200 nucleotides in length that do not encode any protein and can modulate the development of OS in different biological processes (Chen et al., 2021). Some lncRNAs are highly expressed in OS cells and promote tumor proliferation and migration. Recent studies demonstrated that the knockdown of oncogenic lncRNAs not only suppressed the proliferation and promoted the apoptosis of OS cells but also reduced the invasion and migration of OS cells (Zhang et al., 2019; Fei et al., 2019; Li et al., 2019; Lian et al., 2019). LncRNAs act in different ways to reduce OS cell proliferation, including regulating the cell cycle, suppressing cell metabolism, and reducing angiogenesis. However, some lncRNAs are expressed at low levels in OS cells and their overexpression can inhibit tumor development *via* different mechanisms (Wan et al., 2020; Zhou et al., 2020). Most overexpressed lncRNAs suppress tumor progression by targeting miRNAs, although other pathways have also been described. In clinical treatment, chemotherapy resistance is common, resulting in recrudescence and metastases. LncRNAs also play a vital role in chemotherapy resistance through different pathways (Cheng Y et al., 2019; Zhang Q-Q et al., 2021). Various kinds of chemotherapy resistance in OS are related to lncRNAs, and a single lncRNA may be related to multiple kinds of chemotherapy resistance in OS.

**FIGURE 1:**
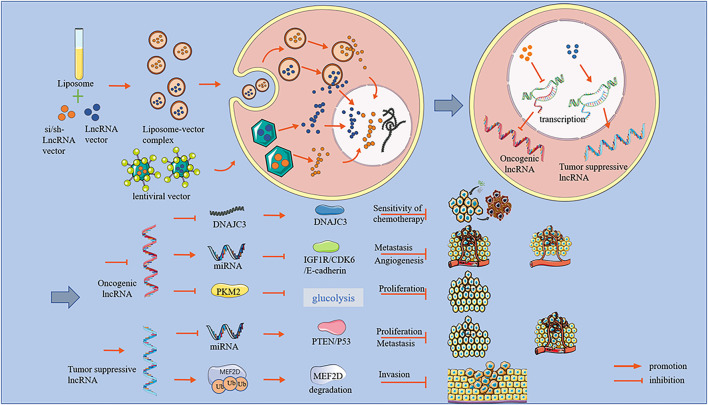
An overview of lncRNA effects and mechanisms.

This review describes recent studies related to lncRNAs and OS and summarizes three main aspects of the potential applications of lncRNAs in the treatment of OS. These aspects are the downregulation of oncogenic lncRNA promoting tumor progression, the upregulation of tumor-suppressive lncRNA, and lncRNA treatments to improve chemotherapy resistance (Figure 1).

## 2 Oncogenic lncRNA silencing inhibits the progression of osteosarcoma

### 2.1 Oncogenic lncRNA silencing inhibits osteosarcoma proliferation and promotes apoptosis

Recent studies have demonstrated that the knockdown of oncogenic lncRNAs can directly inhibit the proliferation of OS cells and promote apoptosis *in vitro*. These data are summarized in Table 1. Li et al. (2019) reported that the upregulation of lncRNA NNT-AS1 promoted OS progression by inhibiting the tumor suppressor miR-320a. They confirmed that lncRNA NNT-AS1 knockdown repressed the proliferation and promoted the apoptosis of OS-732 cells by CCK-8 assays and western blot. Fei et al. (2019) reported that lncRNA ROR functioned as an oncogene in OS by sponging miR‐206. They performed CCK-8 and colony formation assays to demonstrate the inhibition of proliferation and colony formation ability of U2OS cells after lncRNA ROR knockdown. Zheng et al. (2020) proposed that the LINC00266- 1/miR-548c-3p/SMAD2 feedback loop stimulated OS development and showed that lncRNA LINC00266- 1 downregulation partially inhibited MG63 and U2OS cell proliferation by CCK-8 and colony formation assays. In the protein imprinting assay, LINC00266-1 downregulation led to the downregulation of Ki67 and PCNA proteins in OS cells; flow cytometry and TUNEL assays showed that LINC00266-1 knockdown induced OS apoptosis cells. In *in vivo* experiments, LINC00266-1 knockdown inhibited OS growth in nude mice. Zhou et al. (2019) reported that the lncRNA LINC00963 inhibited miR-204-3p by directly binding to it. CCK-8 assay to assess cell growth and viability demonstrated that lncRNA LINC00963 knockdown inhibited OS proliferation by promoting the miR-204-3p/FN1 axis. Luo et al. (2019) found that the lncRNA ADPGK-AS1 affected cell proliferation, invasion, migration, and apoptosis by targeting miR-542-3p in OS. LncRNA ADPGK-AS1 knockout resulted in a significantly decreased cell proliferation rate and protein expression of CDK4 and CyclinD1. TUNEL experiments showed apoptosis induction. Meanwhile, Fei et al. (2020) reported lncRNA AFAP-AS1 as a novel oncogene that promoted OS tumorigenesis and progression by competitively binding miR-497 and regulating IGF1R expression. AFAP1-AS1 knockout significantly inhibited U2OS cell proliferation and colony formation in the CCK-8, plate clone formation, and flow cytometry assays. Chen X et al. (2020) demonstrated that lncRNA BLACAT1 facilitated OS cell growth and motility by upregulating SOX12 through sponging miR-608. They observed reduced 143B and MG63 cell proliferation and increased apoptosis by TUNEL, EdU, and colony formation assays. He et al. (2021) reported that lncRNA CCDC144NL-AS1 promoted OS tumorigenicity by acting as a molecular sponge for microRNA-490-3p, thereby increasing HMGA2 expression. They confirmed through CCK-8 assay and flow cytometry that OS cell proliferation was significantly inhibited and the apoptosis rate was significantly increased after the knockdown of lncRNA CCDC144NL-AS1. *In vivo*, compared to the control group, tumors grew significantly slower and weighed less when lncRNA CCDC144NL-AS1 was silenced. These results indicated that CCDC144NL-AS1 knockdown significantly upregulated the expression of miR-490-3p in HOS and SAOS-2 cells and that CCDC144NL-AS1 expression was positively correlated with HMGA2 mRNA expression in OS tissues. Jin et al. (2021) reported that lncRNA H19 regulated LASP1 expression in OS by competitively binding to miR29a3p. CCK-8 assays showed that RNAH19 knockdown increased miR-29a-3p expression and decreased LASP1 expression, which inhibited Saos2 and MG63 cell proliferation. Huang et al. (2021) confirmed significantly inhibited proliferation and colony formation in U2OS and SAOS-2 cells after lncRNA KCNQ1OT1knockdown, as assessed by CCK-8 assay, colony formation assay, and western blot analysis. The authors suggested that lncRNA KCNQ1OT1 promoted tumor growth and activated the Wnt/β catenin signaling pathway in OS by targeting the miR3666/KLF7 axis. When KCNQ1OT1 was silenced, miR-3666 expression increased significantly. KCNQ1OT1 was negatively correlated with miR-3666 expression. KLF7 expression was negatively correlated with miR-3666 expression and positively correlated with KCNQ1OT1 expression. Jin et al. (2019) reported that lncRNA SND1-IT1 accelerated OS proliferation and migration by upregulating POU2F1 through the sponging of miRNA-665. After knocking down lncRNA SND1-IT1, CCK-8 and EdU assays showed decreased OS cell viability at 48, 72, and 96 h. Compared to the non-knockdown group, the number of colonies formed was significantly reduced and the proportion of EdU-positive cells was lower in the lncRNA SND1-IT1 knockdown group. Zhang Z. F et al. (2019) suggested that lncRNA LINC01116 promoted the proliferation of OS cells by silencing p53 and EZH2. Knockdown of lncRNA LINC01116 significantly reduced OS cell viability and promoted apoptosis, as assessed by CCK8 assay and cell apoptosis determination.

**TABLE 1:** Oncogenic lncRNAs associated with the inhibition of osteosarcoma proliferation and the promotion of apoptosis.

LncRNA	Expression in OS	Regulatory pathway	Key function *in vitro* after knocked-down	Function *in vivo* after knocked-down	Refs
NNT-AS1	↑	MiR-320a/beta-catenin and RUNX2 axis	Proliferation ↓	Tumor formation↓	Li et al. (2019)
			Apoptosis ↑		
			Cell cycle arrested		
ROR	↑	Targeting miR-206	Proliferation ↓	Tumor growth↓	Fei et al. (2019)
			Apoptosis ↑		
00266-1	↑	Linc00266-1/miR-548c-3p/SMAD2 feedback loop	Proliferation↓	Tumor growth↓	Zheng et al. (2020)
			Apoptosis ↑	PCNA and Ki-67 ↓	
			Cell cycle arrested		
00963	↑	miR-204-3p/FN1 axis	Proliferation↓	—	Zhou et al. (2019)
			Apoptosis ↑		
ADPGK-AS1	↑	Targeting miR-542-3p	Proliferation ↓	—	Luo et al. (2019)
			Apoptosis ↑		
			Cell cycle arrested		
AFAP1-AS1	↑	miR-497/IGF1R axis	Proliferation ↓	Tumor growth↓	Fei et al. (2020)
			Apoptosis ↑	Ki-67 expression↓	
BLACAT1	↑	miR-608/SOX12 axis	Proliferation ↓	—	Chen X et al. (2020)
			Apoptosis ↑		
CCDC144NL-AS1	↑	miR-490-3p/HMGA2 axis	Proliferation ↓	Tumor growth↓	He et al. (2021)
			Apoptosis ↑	Tumor volume↓	
				Tumor weight↓	
H19	↑	MiR-29a3p/LASP1 axis	Proliferation↓	Tumor growth↓	Jin et al. (2021)
			Apoptosis ↑	Tumor volume↓	
				Tumor weight↓	
KCNQ1OT1	↑	MiR-3666/KLF7 axis	Proliferation ↓	Tumor volume↓	Huang et al. (2021)
			Apoptosis ↑	Tumor weight↓	
SND1-IT1	↑	miR-665/POU2F1	Apoptosis ↑	—	Jin et al. (2019)
LINC01116	↑	silencing p53 and EZH2	Proliferation ↓	—	Zhang Z. F et al. (2019)
			Apoptosis ↑		
			Cell cycle arrested		

### 2.2 Oncogenic lncRNA silencing could arrest the cell cycle of OS cells

In addition to directly inhibiting OS cell proliferation and apoptosis, lncRNA knockdown could also affect OS cells by regulating the cell cycle. Duan et al. (2019) found that lncRNA MALAT1 exerted an oncogenic function in OS by regulating the miR-34a/CCND1 axis. When lncRNA MALAT1 was silenced, the expression of CCND1, a regulator of the cell cycle, was significantly reduced in Western blot assays. Deng et al. (2019) reported that the lncRNA SNHG1 promoted OS cell proliferation, migration, and invasion by downregulating miRNA-101-3p expression, which enhanced ROCK1 expression. The downregulation of lncRNA SNHG1 induced cell apoptosis and maintained the cell cycle at the G0/G1 phase, thus reducing the overall cell viability. Deng et al. observed that lncRNA LINC00514 promoted OS progression by sponging microRNA-708, thereby increasing the expression of cell proliferation upregulated factor (URGCP). LINC00514 knockdown significantly increased the apoptosis rate of HOS and MG-63 cells; moreover, the proportion of cells in the G0/G1 phase increased according to the proportion of cells in the S phase, as shown by flow cytometry analysis (Yu et al., 2020). Zhang G. F et al. (2021) demonstrated that lncRNA LINC01278 was highly expressed in OS and participated in tumor development by mediating the miR-134-5p/KRAS axis. After LINC01278knockdown, cell cycle analysis showed a significantly increased number of cells in the G1/G0 phase and a significantly decreased number of cells in the S and G2/M phases. These results suggested that LINC01278 knockdown could induce cell apoptosis, arrest the cell cycle at G1/G0, and further inhibit cell proliferation. The mechanism was that miR-134-5p expression increased significantly after LINC01278 knockdown. MiR-134-5p was negatively correlated with LINC01278, and LINC01278 may specifically bind miR-134-5p to participate in OS progression. Therefore, lncRNA LINC01278 could be involved in the regulation of KRAS expression as a sponge of miR-134-5p. Wei et al. (2020) reported that the Usf1-mediated upregulation of lncRNA gAS6-AS2 promoted OS progression through the miR-934/BCAT1 axis. In their study, after AS6-AS2 knockdown, western blot assay showed increased levels of the apoptosis-related molecules caspase 3 and 9, which indicated cell cycle inhibition. Wang et al. (2021) observed that lncRNA HCG9 promoted OS progression through RAD51 by acting as a ceRNA of miR-34b-3p. RAD51 is a key enzyme that regulates the cell cycle and preserves the G2/M phase, whereas H2A preserves cell cycle arrest. After RNAHCG9 knockdown, cell cycle analysis showed that cell arrest was induced, the G0/G1 phase was significantly enhanced, and the G2/M phase was significantly inhibited, thus inhibiting OS progression. After lncRNA SPRY4IT1 knockdown, Yao et al. (2020) demonstrated that cells with cell cycle progression significantly accumulated in the G1 phase, indicating that SPRY4IT1 downregulation could lead to G1 phase arrest. They also found that miR-101 mimic transfection led to S-phase cell cycle arrest. One possible mechanism for these results was that lncRNA SPRY4- IT1 promoted OS progression by regulating the expression of ZEB1 and ZEB2 through miR-101. Shi et al. (2020) proposed that lncRNA AFAP1-AS1 played an oncogenic role in OS through the RhoC/ROCK1/p38MAPK/Twist 1 signaling pathway. Flow cytometry and cell cycle analysis after lncRNA AFAP1-AS1 knockdown showed that compared to the control group, the cell distribution of the knockdown group showed a significant increase in the G0/G1 phase and a significant decrease in the S phase; however, the two groups did not differ significantly in the G2/M phases. In summary, the cell cycle was arrested in the G0/G1 phase, and S cell cycle progression was inhibited after AFAP1-AS1 knockdown. Wang and Zhang (2021) observed that lncRNA CASC15 promotes IS proliferation and metastasis by regulating the epithelial-mesenchymal transition *via* the Wnt/β-catenin signaling pathway. CASC15 affected the cell cycle by activating the Wnt/β-catenin pathway, thereby promoting cell proliferation. After CASC15 knockdown, CCK-8 and EdU assays showed decreased OS cell proliferation. The number of cells in the G0/G1, S, and G2/M phases detected by flow cytometry showed a decreased number of cells in the S phase. The effect of lncRNA knockdown on cell metabolism may also have an inhibitory effect on OS progression. Pan et al. (2022) reported that lncRNA HCG18 promoted OS growth by enhancing aerobic glycolysis *via* the miR-365a-3p/PGK1 axis. HCG18 knockdown partly reduced the extracellular acidification rate (ECAR) in MNNG-HOS and 143B cells but enhanced the oxygen consumption rate (OCR) of these cells. The results of the cell metabolism experiment showed decreased glucose consumption, increased ATP levels, and decreased lactate production in OS cells when lncRNA HCG18 was silenced. These results indicated that HCG18 knockdown inhibited aerobic glycolysis in OS cells, thereby inhibiting tumor cell growth. Additionally, Wang et al. (2020b) found that lncRNA OR3A4 regulated OS cell growth by modulating miR-1207-5p/G6PD signaling. Glucose consumption assays using MG-63 and SaoS-2 cells with lncRNA OR3A4 knockdown showed significantly reduced NADPH levels and inhibited glucose consumption and lactate production in OS cells. These results suggested that OR3A4 knockdown suppressed glycolysis in OS cells. Chang et al. (2021) reported inhibited OS cell metabolism following the downregulation of lncRNA PCAT-1. Because lncRNA PCAT-1 promoted OS progression through the mir-508-3p/ZEB1 axis, miR-508-3p levels increased significantly after lncRNA PCAT-1 knockdown. miR-508-3p expression was negatively correlated with pCAT-1. LncRNA PCAT-1 knockdown reduced ZEB1 expression, thereby inhibiting OS progression. These data are summarized in Table 2.

**TABLE 2:** Oncogenic lncRNAs associated with the cell cycle arrest of osteosarcoma cells.

LncRNA	Expression in OS	Regulatory pathway	Key function *in vitro* after knocked-down	Function *in vivo* after knocked-down	Refs
MALAT1	↑	miR-34a/CCND1 axis	Proliferation ↓	—	Duan et al. (2019)
			Apoptosis↑		
			Cell cycle arrested		
SNHG1	↑	miR-1013p/ROCK1 axis	Proliferation ↓	—	Deng et al. (2019)
			Apoptosis ↑		
			Cell cycle arrested		
00514	↑	LINC00514/miR-708/URGCP pathway	Proliferation ↓	Tumor volume↓	Yu et al. (2020)
			Apoptosis ↑	Tumor weight↓	
			Cell cycle arrested		
LINC01278	↑	miR-134-5p/KRAS axis	Proliferation↓	Tumor volume↓	Zhang G. F et al. (2021)
			Apoptosis ↑	Tumor weight↓	
			Cell cycle arrested		
GAS6-AS2	↑	miR-934/BCAT1 axis	Proliferation ↓	Tumor volume↓	Wei et al. (2020)
			Apoptosis ↑	Tumor weight↓	
			Cell cycle arrested		
HCG9	↑	miR-34b-3p/RAD51 axis	Proliferation ↓	Tumor volume↓	Wang et al. (2021)
			Apoptosis↑	Tumor weight↓	
			Cell cycle arrested	Cell cycle arrested	
SPRY4-IT1	↑	miR-101/ZEB1 and ZEB2 axis	Proliferation ↓	Tumor volume↓	Yao et al. (2020)
			Apoptosis ↑	Tumor weight↓	
			Cell cycle arrested	E-cadherin ↑	
			EMT ↓		
AFAP1-AS1	↑	RhoC/ROCK1/p38MAPK/Twist1 pathway	Proliferation↓	Tumor volume↓	Shi et al. (2020)
			Apoptosis ↑	Tumor weight↓	
			Cell cycle arrested		
			Angiogenesis↓		
			EMT ↓		
CASC15	↑	Wnt/β-catenin pathway	Proliferation ↓	Tumor volume↓	Wang and Zhang (2021)
			Apoptosis ↑		
			Cell cycle arrested		
			EMT ↓		
HCG18	↑	miR-365a-3p/PGK1 axis	Proliferation ↓	Tumor growth↓	Pan et al. (2022)
			Apoptosis↑	Tumor volume↓	
			Cell metabolism ↓	Tumor weight↓	
				Ki-67 expression↓	
OR3A4	↑	miR-1207-5p/G6PD axis	Proliferation ↓	—	Wang et al. (2020b)
			Apoptosis ↑		
			Cell metabolism↓		
PCAT-1	↑	miR-508-3p/ZEB1 axis	Proliferation ↓	—	Chang et al. (2021)
			Apoptosis ↑		

### 2.3 Oncogenic lncRNA silencing reduces osteosarcoma angiogenesis

Inhibition of OS cell proliferation is also related to the regulation of angiogenesis by lncRNAs. Xiao et al. (2020) reported that LINC00265 targets miR-382-5p and regulates SAT1, VAV3, and angiogenesis in OS. They demonstrated inhibited cell tube production capacity *via* LINC00265 silencing. Therefore, LINC00265 knockdown caused a decreased ability to form blood vessels. Vimalraj et al. (2021) hypothesized that lncRNA MALAT1 promotes tumor angiogenesis by regulating microRNA-150-5p/VEGFA signaling in OS. They analyzed the levels of angiogenin-related genes and VEGFA by Western blot and ELISA and found that lncRNA MALAT1 knockdown significantly reduced the expression and secretion levels of VEGFA in MG63 and SaOS2 cells. Using chicken embryos, they established a new model to examine the regulation of angiogenesis in tumors. Compared to the control group, the MALAT1 knockdown group showed reduced blood vessel length and size and connections between blood vessels, as well as decreased mRNA expression levels of the angiogenic markers VEGFA, ANG1, Tie2, CXCR4, and FGF2. LncRNA AFAP1-AS1 is overexpressed in OS and plays an oncogenic role through the RhoC/ROCK1/p38MAPK/Twist-1 signaling pathway. Shi et al. (2019) reported that AFAP1-AS1 knockdown reduced vasculogenic mimicry in OS cells. Vasculogenic mimicry (VM) is the formation of microvascular structures in malignant tumors and is believed to be closely related to cancer cell growth, invasion, and metastasis. To investigate whether AFAP1-AS1 affects VM formation in OS cells, the authors performed a tube formation assay. Compared to the control group, the AFAP1-AS1 knockdown group showed a significantly reduced number of tubular structures in OS cells, indicating that AFAP1-AS1 knockdown could inhibit VM formation and that AFAP1-AS1 may play an important role in VM formation in OS cells. Chen et al. (2021b) demonstrated that the LOC100129620/miR-335-3p/CDK6 signaling pathway promotes OS metastasis. The results of cell scratch experiments showed that the knockdown of lncRNA LOC100129620 reduced the stimulating effect of OS cells on endothelial cell migration. Similarly, LOC100129620 knockdown reduced the number of endothelial cells within the xenograft model. These results indicated that LOC100129620 knockdown can inhibit OS angiogenesis. Thus, lncRNAs play an important regulatory role in various pathophysiological processes of OS, including cell proliferation, cell cycle progression, apoptosis, and carcinogenesis. These data are summarized in Table 3.

**TABLE 3:** Oncogenic lncRNAs associated with reduced osteosarcoma angiogenesis.

LncRNA	Expression in OS	Regulatory pathway	Key function *in vitro* after knocked-down	Function *in vivo* after knocked-down	Refs
00265	↑	targeting miR-382-5p	Proliferation ↓	Tumor growth↓	Xiao et al. (2020)
			Apoptosis ↑	Tumor volume↓	
			Angiogenesis↓	SAT1, VAV3↓	
LncRNA MALAT1	↑	miRNA-150-5p/VEGFA axis	Proliferation↓	Angiogenesis ↓	Vimalraj et al. (2021)
			Apoptosis ↑		
			Angiogenesis↓		
AFAP1-AS1	↑	RhoC/ROCK1/p38MAPK/Twist1	Proliferation ↓	Tumor growth↓	Shi et al. (2019)
			Angiogenesis↓	Tumor volume↓	
LOC100129620	↑	CDK6 expression	Proliferation ↓	Tumor growth↓	Chen et al. (2021b)
			Apoptosis ↑	Tumor volume↓	
			Cell cycle arrested	Angiogenesis ↓	
			Angiogenesis↓		

### 2.4 Oncogenic lncRNA silencing suppresses invasion and migration in OS

An increasing number of studies have revealed that the knockdown of oncogenic lncRNAs can suppress OS development, especially cell invasion and cell migration. These data are summarized in Table 4. Zhang C et al. (2019) reported that lncRNA MIAT might function as a sponge of miR-128-3p in OS and that the invasion and migration of MG63 cells after downregulation of lncRNA CMIAT were inhibited by transwell and wound healing assays, respectively. Meanwhile, Lian et al. (2019) confirmed that lncRNA LINC00460 knockdown remarkably repressed OS cell invasion and migration by transwell and wound healing assays; they also reported that Linc00460 functioned as a competitively endogenous RNA (ceRNA) by sponging miR-1224-5p in OS, thereby promoting OS progression. LncRNA LINC00665 facilitated OS progression by reducing miR-3619 expression. Zhang D. W et al. (2020) verified that LINC00665 knockdown suppressed OS cell invasion and migration in transwell assays. Xing et al. (2020) confirmed that upregulated LINC00689 competitively bound to miR-655, which prevented SOX18 from miRNA-mediated degradation, thus facilitating OS progression. As evidenced by wound healing and transwell assays, when LINC00689 was silenced, the migratory and invasive abilities of MG63 and 143B cells were notably impaired. Moreover, transwell and wound healing assays to investigate the role of lncRNA LINC01614 in the regulation of cell migration and invasion in OS cells demonstrated reduced cell migration and invasion ability in the LINC016141 knockdown group compared to the control group. Additionally, Cai et al. (2021) demonstrated that lncRNA LINC01614 could function as a competing endogenous RNA and promote OS cell proliferation and invasion through the miR-520a-3p/SNX3 axis. Zhang W et al. (2020) reported that the downregulation of lncRNA DANCR inhibited OS cell migration and metastasis and proposed a mechanism in which lncRNA DANCR regulates OS migration and invasion by targeting the miR-49/MSI2 axis. Dai et al. (2021) reported that the lncRNA EBLN3P promoted OS *via* the miR-224-5p/Rab10 regulatory loop. When lncRNA EBLN3P was knocked down, transwell and wound healing experiments with OS cells showed inhibited invasion and migration by OS cells. Zheng and Lin (2021) found that lncRNA HCG18 promotes OS progression through miR-148b/ETV5 regulation; a transwell assay in OS cells after LncRNAHCG18 knockdown showed significantly suppressed migration and invasion. Wang et al. (2019) proposed that lncRNA HIF1A-AS2 overexpression promoted OS progression through the modulation of miR-129-5p. The authors used wound healing and invasion assays of OS cells measure determine cell proliferation and invasion ability and concluded that silencing lncRNA HIF1A-AS2 significantly decreased OS cell invasion and metastasis. In summary, many experiments have shown that the knockout of specific lncRNAs can inhibit OS cell invasion and migration.

**TABLE 4:** Oncogenic lncRNAs associated with osteosarcoma invasion and migration.

LncRNA	Expression in OS	Regulatory pathway	Key function *in vitro* after knockdown	Function *in vivo* after knockdown	Refs
MIAT	↑	MIAT/miR-128-3p/VEGFC axis	Invasion ↓	—	Zhang C et al. (2019)
			Migration ↓		
			VEGFC ↑		
00460	↑	00460/miR-1224-p/FADS1 axis	Invasion ↓	—	Lian et al. (2019)
			Migration ↓		
00665	↑	sponging miR-3619	Invasion ↓	—	Zhang D. W et al. (2020)
			Migration ↓		
00689	↑	miR-655/SOX18 axis	Invasion ↓	—	Xing et al. (2020)
			Migration ↓ caspase3/9 ↑		
01614	↑	miR-520a-3p/SNX3 axis	Invasion ↓	—	Cai et al. (2021)
			Migration ↓		
DANCR	↑	miR-149/MSI2 axis	Invasion ↓	—	Zhang W et al. (2020)
			Migration ↓		
BLN3P	↑	miR-224-5p/Rab10 axis	Invasion ↓	—	Dai et al. (2021)
			Migration ↓		
HCG18	↑	miR-148b/ETV5	Invasion ↓	—	Zheng and Lin (2021)
		axis	Migration ↓		
HIF1A-AS2	↑	Sponging miR-129-5p	Invasion ↓	—	Wang et al. (2019)
			Migration ↓		

### 2.5 Oncogenic lncRNA regulates the expression of EMT-related proteins in OS cells

The epithelial-interstitial transformation (EMT) is a basic process that controls the morphogenesis of organisms. EMT manifests as a loss of E-cadherin and an increase in vimentin expression and is a process in which epithelial cells lose their polarity and adhesion to gain migratory ability and adopt a mesenchymal phenotype. Knockdown of lncRNAs can also regulate the expression of EMT-related proteins in OS cells. LINC00324 accelerated OS proliferation and migration by regulating WDR66. Wu et al. (2020) used western blot analysis to evaluate the effect of LINC00324 on EMT progression. After the knockdown of LINC00324 in OS cells, the expression level of E‐cadherin increased whereas the expression levels of N‐cadherin and Vimentin decreased. Yan et al. (2021) reported that LINC00467 serves as a molecular sponge for miR-217, while karyopherin subunit α4 (KPNA4) is a downstream target gene of miR-217; LINC00467 facilitates OS progression by sponging miR-217 to regulate KPNA4 expression. The authors reported decreased expression levels of EMT-associated proteins after LINC00467 silencing, as assessed by western blot analysis. In contrast, for the same lncRNA LINC00467, Ma et al. (2020) concluded that lncRNA LINC00467 contributes to OS growth and metastasis by regulating HMGA1 by directly targeting miR-217. In their western blot analysis, lncRNA LINC00467 downregulation increased E-cadherin expression and decreased N-cadherin and vimentin expression. Zhou and Mu (2021) also reported that the results of Western blot analysis showed that LINC00958 knockdown downregulated N-cadherin and vimentin expression while upregulating E-cadherin expression. The authors concluded that LINC0095 promotes tumorigenesis and metastasis in OS by competitively inhibiting miR-4306 expression, leading to elevated CEMIP expression. Gu Z. Q et al. (2020) reported that lncRNA LINC01419 mediated malignant phenotypes in OS by targeting the miR-519a-3p/PDRG1 axis and that LINC01419 downregulation increased the expression of E-cadherin protein while lowering the expression levels of N-cadherin, vimentin, and β-catenin. Zhang H et al. (2020) demonstrated that DDX11-AS1 could sponge miR-873-5p to upregulate DDX11 expression and that DDX11-AS1 contributed to OS progression by stabilizing DDX11. Meanwhile, western blot analysis indicated that DDX11-AS1 deficiency markedly reduced MMP2, MMP9, N-cadherin, Slug, and Twist expression but increased E-cadherin expression, suggesting that silencing DDX11-AS1 could suppress cell metastasis and the EMT process. Additionally, Wei et al. (2020) proposed that USF1-mediated upregulation of lncRNA GAS6-AS2 facilitated OS progression through the miR-934/BCAT axis. They measured the expression levels of EMT-relevant molecules, N-cadherin, E-cadherin, and vimentin in OS cells after GAS6-AS2 knockdown. The data demonstrated that GAS6-AS2 depletion dramatically reduced N-cadherin and vimentin levels, while GAS6-AS2 silencing significantly increased E-cadherin levels. Liu et al. (2021) reported that lncRNA X-inactive (XIST) promoted OS metastasis by modulating microRNA-758/Rab 16. The results of Western blot analysis showed that transfection with si-XIST upregulated E-cadherin at the protein level while downregulating the protein levels of N-cadherin and vimentin in OS cells. In brief, EMT is an important way to promote OS invasion and migration and enhance its malignant behaviors. Knockdown of specific lncRNAs could inhibit EMT progression and delay tumor invasion and metastasis by reducing N-cadherin and vimentin levels while enhancing E-cadherin expression. These data are summarized in Table 5.

**TABLE 5:** Oncogenic lncRNA associated with the regulation of EMT-related protein expression in osteosarcoma cells.

LncRNA	Expression in OS	Regulatory pathway	Key function *in vitro* after knockdown	Function *in vivo* after knockdown	Refs
00324	↑	Regulating WDR66	Invasion ↓	—	Wu et al. (2020)
			Migration ↓		
			E‐cadherin ↑		
			N‐cadherin ↓		
			Vimentin ↓		
00467	↑	miR-217/KPNA4 axis	Invasion ↓	—	Yan et al. (2021)
			Migration ↓		
			E‐cadherin ↑		
			N‐cadherin ↓		
			Vimentin ↓		
00467	↑	miR-217/HMGA1 axis	Invasion ↓	—	Ma et al. (2020)
			Migration ↓		
			E‐cadherin ↑		
			N‐cadherin ↓		
			Vimentin ↓		
00958	↑	miR-4306/CEMIP axis	Invasion ↓	—	Zhou and Mu (2021)
			Migration ↓ caspase-3 ↑ E‐cadherin ↑		
			N‐cadherin ↓		
			Vimentin ↓		
01419	↑	miR-519a-3p/PDRG1 axis	Invasion ↓	Tumor volume↓	Gu Z. Q et al. (2020)
			Migration ↓	PCNA/Ki-67 ↓	
			E‐cadherin ↑		
			N‐cadherin ↓		
			Vimentin ↓		
			β-catenin ↓		
DDX11-AS1	↑	Stabilizing DDX11	Invasion ↓	Tumor volume↓	Zhang H et al. (2020)
			Migration ↓	Tumor weight↓	
			E‐cadherin ↑		
			N‐cadherin ↓		
			Vimentin ↓		
GAS6-AS2	↑	miR-934/BCAT1 axis	Invasion ↓	—	Wei et al. (2020)
			Migration ↓		
			E‐cadherin ↑		
			N‐cadherin ↓		
			Vimentin ↓ caspase-3 ↑		
			caspase-9 ↑		
X-inactive	↑	microRNA-758/Rab16 axis	Invasion ↓	—	Liu et al. (2021)
			Migration ↓		
			E‐cadherin ↑		
			N‐cadherin ↓		
			Vimentin ↓		

### 2.6 Oncogenic lncRNA silencing demonstrates antitumor effects *in vivo*

As described above, the knockdown of oncogenic lncRNAs significantly inhibited the progression of OS cells *in vitro*. Furthermore, several studies have shown similar results *in vivo*. Researchers typically transferred OS cells with silenced oncogenic lncRNAs into mice and observed tumor cell growth to determine the role of lncRNAs *in vivo*. Ning and Bai (2021) injected MG63 cells after DSCAM-AS1 silencing subcutaneously into the groin of each mouse. The results showed decreased tumor size and weight in the DSCAM-AS1-silenced group after 4 weeks. They proposed that DSCAM-AS1 accelerated OS cell proliferation and migration through miR-186-5p/GPRC5A signaling. Li et al. (2021) reported that FGD5-AS1 increased OS cell proliferation and migration by sponging miR-506-3p to upregulate RAB3D. They injected OS cells transfected with pcDNA3.1-FGD5-AS1 or FGD5-AS1 siRNA into nude mice *via* the caudal vein, in which FGD5-AS1 overexpression promoted the lung metastasis of OS cells, while FGD5-AS1 depletion repressed the formation of metastatic nodules in the lung. Li et al. (2020) established an OS mouse model to study the effects of lncRNA HULC on OS cell invasion and metastasis, and observed smaller tumor tissues and fewer metastatic tumors in the HULC knockdown group compared to those in the control group. Finally, lncRNA HULC induces OS progression by regulating the miR-372-3p/HMGB1 signaling axis. Chen et al. (2021a) confirmed that lncRNA NEAT1 promoted the epithelial-mesenchymal transition and metastasis of OS cells by sponging miR-483 to upregulate STAT3 expression *in vivo*. They observed significantly smaller tumor volumes, lighter tumor weights, slower tumor growth rates, and fewer metastatic tumors in the lncRNA NEAT1-silenced group compared to those in the control group. Liu W et al. (2020) found that lncRNA PGM5-AS1 silencing inhibited OS cell tumorigenesis and reduced the number of lung metastases in a mouse tumor model. They suggested that lncRNA PGM5-AS1 promoted the epithelial-mesenchymal transition, invasion, and metastasis of OS cells by impairing miR-140-5p-mediated FBN1 inhibition. Jiang et al. (2021) injected OS cells into mice to construct an OS model *in vivo*. Six weeks later, compared to the control group, the lncRNA RUSC1-AS1 knockdown group showed significantly reduced tumor volume; attenuated tumor node weight; increased E-cadherin expression; and decreased N-cadherin, vimentin, and Snail expression. They demonstrated that lncRNA RUSC1-AS1 functioned as a competing endogenous RNA (ceRNA) to competitively bind miR-101-3p, thus upregulating Notch 1 expression and mediating the malignant behaviors of OS cells. Yu et al. (2019) proposed that the lncRNA taurine promoted OS cell metastasis by mediating HIF-1α *via* miR-143-5p. In their tumor mouse model, compared to the control group, the lncRNA TUG1 knockdown group showed significantly less visible peritoneal and pulmonary nodules and smaller tumor volumes and weights. Ding et al. (2021) reported the results of a tail vein injection lung metastasis model, in which the number of pulmonary metastasis nodules formed by OS cells in the MELTF-AS1-silenced group (shRNA MELTF-AS1) was significantly reduced compared to that in the control group. Additionally, the survival time of mice in the MELTF-AS1-silenced group was longer than that in the control group. Immunofluorescence assays revealed that after silencing MELTF-AS1 in OS cells, the protein level of Vimentin decreased, and the protein level of E-cadherin increased. These results suggested a weakened metastasis ability of OS cells *in vivo* after MELTF-AS1 silencing. Indeed, MELTF-AS1 adsorbed miR-485-5p in the cytoplasm and acted as a ceRNA to promote MMP14 expression. In general, lncRNA plays an important role in OS progression *in vivo*; the downregulation of specific lncRNA significantly inhibited tumor growth, invasion, and metastasis in transfection and tail vein injection experiments in mice. These data are summarized in Table 6.

**TABLE 6:** Oncogenic lncRNAs associated with antitumor effects *in vivo*.

LncRNA	Expression in OS	Regulatory pathway	Key function *in vitro* after knockdown	Function *in vivo* after knockdown	Refs
DSCAM-AS1	↑	miR-186-5p/GPRC5A axis	Invasion ↓	Tumor volume↓	Ning and Bai (2021)
			Migration ↓	Tumor weight↓	
			E‐cadherin ↑	E‐cadherin ↑	
			N‐cadherin ↓	N‐cadherin ↓	
			Vimentin ↓	Vimentin ↓	
FGD5-AS1	↑	miR-506-3p/RAB3D axis	Invasion ↓	lung metastasis ↓	Li et al. (2021)
			Migration ↓		
HULC	↑	miR-372-3p/HMGB1 axis	Invasion ↓	Tumor volume↓	Li et al. (2020)
			Migration ↓	Tumor weight↓ lung metastasis ↓	
NEAT1	↑	miR-483 to upregulate STAT3 expression	Invasion ↓	Tumor volume↓	Chen et al. (2021a)
			Migration ↓	Tumor weight↓ lung metastasis ↓	
			E‐cadherin ↑	Liver metastasis ↓	
			N‐cadherin ↓	E‐cadherin ↑	
				N‐cadherin ↓	
				Vimentin ↓	
			Vimentin ↓		
PGM5-AS1	↑	miR-140-5p/FBN1 axis	Invasion ↓	Tumor volume↓	Liu W et al. (2020)
			Migration ↓	Tumor weight↓ lung metastasis ↓	
			E‐cadherin ↑	E‐cadherin ↑	
			N‐cadherin ↓	N‐cadherin ↓	
			Vimentin↓	Vimentin ↓	
RUSC1-AS1	↑	Notch1 pathway	Invasion ↓	Tumor volume↓	Jiang et al. (2021)
			Migration ↓	Tumor weight↓ lung metastasis ↓	
			E‐cadherin ↑	E‐cadherin ↑	
			N‐cadherin ↓	N‐cadherin ↓	
			Vimentin ↓	Vimentin ↓	
Taurine	↑	miR-143-5p/HIF-1α axis	Invasion ↓	Tumor volume↓	Yu et al. (2019)
			Migration ↓	peritoneal spread ↓	
			Angiogenesis ↓		
			E‐cadherin ↑		
			N‐cadherin ↓		
			Vimentin ↓		
MELTF-AS1	↑	miR-485-5p/MMP14 axis	Invasion ↓	Tumor volume↓	Ding et al. (2021)
			Migration ↓	Tumor weight↓ lung metastasis ↓	
			E‐cadherin ↑		
			N‐cadherin ↓		
			Vimentin ↓		

## 3 Tumor-suppressive lncRNA overexpression inhibits OS progression

According to different regulatory mechanisms of lncRNAs in OS progression, not only the knockdown of oncogenic lncRNAs but also the overexpression of tumor-suppressive lncRNAs could inhibit OS progression. These data are summarized in Table 7.

**TABLE 7:** Tumor suppressive lncRNAs associated with the inhibited progression of osteosarcoma targeting miRNA.

LncRNA	Expression in OS	Regulatory pathway	Key function after overexpression	Refs
LINC00588	↓	miR-1972/TP53 axis	Proliferation↓	Zhou et al. (2020)
			Invasion ↓	
			Migration ↓	
			Cell cycle arrested	
			E‐cadherin ↑	
			N‐cadherin ↓	
			Vimentin ↓	
LINC00691	↓	miR-1256/ST5 axis	Proliferation↓	Wan et al. (2020)
			Invasion ↓	
			E‐cadherin ↑	
GAS5	↓	miR-23a-3p/PI3K/AKT pathway	Proliferation ↓	Liu J et al. (2020)
			Invasion ↓	
			Apoptosis ↑	
HCG11	↓	miR-942-5p/p27Kip1 axis	Proliferation ↓	Gu et al. (2021)
			Cell cycle arrested	
NR_027471	↓	miR-8055/TP53INP1 axis	Proliferation↓	Chen J et al. (2020)
			Invasion ↓	
			Migration ↓	
TUSC7	↓	miR-181a/RASSF6 axis	Proliferation ↓	Zhao et al. (2021)
			Invasion ↓	
			Apoptosis ↑	

### 3.1 Tumor-suppressive lncRNAs can suppress OS by targeting miRNAs

Tumor-suppressive lncRNAs always inhibit OS progression by affecting miRNAs. The expression level of lncRNA LINC00588 is low in HOS and U2OS cell lines. Zhou et al. (2020) transfected these cell lines with the lentiviral vector pLVX-LINC00588. The results of CCK-8, wound healing, and transwell assays showed inhibition of cell viability, migration, and invasion, respectively, due to LINC00588 overexpression. Cell cycle analysis with a cell cycle analysis kit showed arrested cell cycles in the G2 phase in OS. Furthermore, to assess tumor cell metastasis ability, the expression of EMT in transfected cells showed upregulated expression of E-cadherin and downregulated expression of ZEB1, Snail, and Fibronectin. Finally, the authors demonstrated that lncRNA LINC00588 was a ceRNA for miRNA-1972 and inhibited TP53 expression. Wan et al. (2020) overexpressed lncRNA LINC00691 *via* pLVX-IRES-Puro vectors in U2OS and Saos-2 cell lines. They observed reduced proliferation and invasion of OS cells overexpressing lncRNA LINC00691 in CCK-8 analysis and transwell assay, respectively. They also demonstrated increased E-cadherin expression and decreased ZEB1, snail, and fibronectin expression with lncRNA LINC00691 overexpression in EMT analysis. Finally, they demonstrated that lncRNA LINC00691 acts as a ceRNA for miRNA-1256 and promotes ST5 expression. LncRNA GAS5 has been identified as a tumor suppressor in several human cancers involving OS. Liu J et al. (2020) transfected U2OS and Saos-2 cell lines with the pcDNA-GAS5 vector, in which GAS5 overexpression not only inhibited OS cell proliferation and invasion in CCK-8 and transwell assays but also promoted apoptosis, as shown by flow cytometry. LncRNA GAS5 acted as a ceRNA for miR-23a-3p and inhibited the activation of the PI3K/AKT pathway. Finally, in their tumor xenograft BALB/c nude mouse model, compared to the pcDNA3.1 empty vector group, the pcDNA-GAS5 vector group showed a smaller tumor size and a lower miR-23a-3p expression level. HCG11 is another lncRNA with decreased expression in OS cells. Gu et al. (2021) transfected MG63 and 143B cell lines with the lncRNA HCG11 vector and reported that HCG11 overexpression decreased OS cell proliferation in CCK-8 assays; moreover, the cell cycle was arrested in cell cycle analysis, and DNA replication activity was inhibited in EdU assays. *In vivo*, a BALB/c nude mouse tumor model was established. Compared to the control group, tumor volumes were smaller in the HCG11 overexpression group. Finally, the authors demonstrated that lncRNA HCG11 works by binding to miR-942-5p and IGF2BP2 and increasing p27Kip1 expression. Chen J et al. (2020) reported that lncRNA NR_027471 suppresses OS both *in vitro* and *in vivo*. After transfecting the NR_027471 vector into U2OS and Saos-2 cell lines, inhibition of proliferation, migration, and invasion were observed in the CCK-8 assay, scratch test, and transwell assay respectively. A nude mouse tumor xenograft model was established, which showed a significantly lower tumor weight in the NR_027471 group compared to that in the control group. The authors also confirmed the regulatory mechanism by which lncRNA NR_027471 upregulates TP53INP1 by sponging miR-8055. After observing significantly downregulated lncRNA TUSC7 expression in OS tissues, Zhao et al. (2021) transfected U2OS and MG63 cells with pcdNA3. 1-TUSc7 (a TUSc7 overexpression vector). CCK-8 assay, colony formation assay, transwell assay, and flow cytometry analysis, showed lncRNA TUSC7 overexpression inhibited OS cell proliferation and invasion and promoted their apoptosis *in vitro*. *In vivo*, a BALB/c nude mouse tumor xenograft model was established, which showed significantly smaller tumors in the TUSc7-overexpressing group. Finally, they demonstrated that lncRNA TUSC7 acted as a ceRNA for miR-181a and upregulated RASSF6 expression. These data are summarized in Table 7.

### 3.2 Tumor-suppressive lncRNAs inhibit OS by mechanisms other than miRNAs

In addition to targeting miRNAs, overexpressed lncRNAs can suppress OS *via* other methods. Zhao et al. (2019) transfected U2OS and MG63 cells with pcDNA-EPIC1, in which the lncRNA EPIC1 level was significantly higher than that of the control group. The results of the MTT assay and BALB/c nude mouse xenograft model showed inhibited cell viability and suppressed tumor growth, which indicated that OS progression could be inhibited by lncRNA EPIC1 overexpression. Their research also indicated that the effect of lncRNA EPIC1 was mediated by promoting the ubiquitylation of MEF2D. The authors observed decreased lncRNA HIF2PUT expression in U2OS and MG-63 stem cell lines and transfected these cells with pcDNA-HIF2PUT. The results of the wound-healing and transwell assays suggested that a high lncRNA HIF2PUT level inhibited OS cell migration and invasion. These results were reversed in the HIF2PUT knockdown groups. Finally, the inhibitory effect of lncRNA HIF2PUT was attributed to a positive correlation between lncRNA HIF2PUT and HIF-2a (Ding et al., 2019). Xin et al. (2022) transfected OS cells isolated from frozen OS tissue with the lncRNA LINC00619 vector. In addition to the inhibition of OS cell proliferation, migration, and invasion by LINC00619 overexpression in the MTT assay, scratch test, and transwell assay, respectively, LINC00619 overexpression also promoted OS apoptosis in flow cytometry analysis. Furthermore, the PI3K/Akt pathway and HGF expression were repressed in OS cells overexpressing LINC00619. The tumor-suppressive effect of lncRNA H19 was verified *in vitro* and *in vivo*. Xu et al. (2020) infected U2OS cells with a virus containing H19, and H19-bound protein complexes were identified by mass spectrometry. Wiki pathway and GO_BP analyses showed that these putative H19-associated protein complexes mainly functioned in DNA damage response, DNA repair, and the cell cycle. These findings indicated that lncRNA H19 suppresses OS genesis through DNA repair protein complexes. To determine the effects of lncRNA MEG3 on OS progression, Chen L et al. (2020) transfected MG-63 cells with lncRNA MEG3 vectors. As a result, the cell proliferation of the MEG3 group was significantly inhibited in the CCK-8 assay; the cell apoptosis of the MEG3 group was remarkably promoted in Hoechst 33258 staining, and the expression of the apoptosis-related protein Caspase3 in the MEG3 group was markedly increased in western blotting. Furthermore, they reported that lncRNA MEG3 overexpression inhibited the Notch signaling pathway in OS cells. Huang et al. (2022) prepared c(RGDyK)-modified and MEG3-loaded exosomes (cRGD-Exo-MEG3) and proved that they could be delivered more efficiently to OS cells both *in vitro* and *in vivo*. In OS cells targeted by cRGD-Exo-MEG3, cell proliferation was inhibited in CCK-8 and colony formation assays; cell invasion ability was suppressed in transwell assays, and cell apoptosis was promoted in flow cytometry analysis. *In vivo*, a xenograft tumor model was applied, in which cRGD-Exo-MEG3 treatment markedly reduced the tumor volume compared to that of the control groups. These results indicated that lncRNA MEG3-loaded exosomes may be an effective therapy to suppress OS. These data are summarized in Table 8.

**TABLE 8:** Tumor suppressive lncRNAs associated with osteosarcoma inhibition by mechanisms other than miRNAs.

LncRNA	Expression in OS	Regulatory pathway	Key Function after overexpression	Refs
EPIC1	↓	MEF2D ubiquitylation	Proliferation ↓	Zhao et al. (2019)
HIF2PUT	↓	Promoting HIF-2a	Invasion ↓	Ding et al. (2019)
			Migration ↓	
LINC00619	↓	PI3K/Akt and HGF inhibition	Proliferation↓	Xin et al. (2022)
			Invasion ↓	
			Migration ↓	
H19	↓	DNA repair protein complexes	Cell cycle arrested	Xu et al. (2020)
MEG3	↓	Notch pathway inhibition	Proliferation↓	Chen L et al. (2020)
			Apoptosis ↑	
MEG3	↓	—	Proliferation↓	Huang et al. (2022)
			Invasion ↓	
			Apoptosis ↑	

## 4 LncRNA treatment improves chemotherapy resistance

Although obvious improvements have been made, chemotherapy resistance remains a major obstacle causing recrudescence and metastases. Recent studies have also demonstrated the vital role of lncRNAs in OS chemotherapy resistance, as shown in Table 9.

**TABLE 9:** LncRNAs associated with improved chemotherapy resistance.

LncRNA	Expression in OS	Regulatory pathway	Affected chemotherapy-resistance types	Refs
FOXD2-AS1	↑	Targeting miR-143	Cisplatin	Zhang Q-Q et al. (2021)
NCK-AS1	↑	Targeting miR-137	Cisplatin	Cheng Y et al. (2019)
ROR	↑	Sponging miR-153-3p	Cisplatin	Cheng F-H et al. (2019)
SARCC	↓	upregulating miR-143	Cisplatin	Wen et al. (2020)
LINC00922	↑	TFAP2C/LINC00922/miR-424-5p	Doxorubicin	Gu Z et al. (2020)
lncARSR	↑	AKT/MRP1 axis	Doxorubicin	Shen and Cheng (2020)
Sox2OT-V7	↑	Targeting miR-142 and miR-22	Doxorubicin	Zhu et al. (2020)
ANCR	↑	—	Doxorubicin	Hu et al. (2021)
LAMTOR5-AS1	↑	Regulating NRF2	Etoposide	Pu et al. (2021)
			Carboplatin	
			Cisplatin	
OIP5-AS1	↑	sponging miR-137-3p	Doxorubicin	Sun et al. (2020)
OIP5-AS1	↑	miR-377-3p/FOSL2 axis	Cisplatin	Liu and Wang (2020)

### 4.1 LncRNAs could be treatment targets to suppress cisplatin resistance in OS

Various lncRNAs can sensitize OS cells to cisplatin *via* knockdown or overexpression. Zhang Q-Q et al. (2021) reported that lncRNA FOXD2-AS1 knockdown inhibited miR-143 expression to inhibit OS resistance to cisplatin. They transfected siRNA targeting FOXD2-AS1 into cisplatin-resistant OS cells. The CCK-8 assay showed a significantly lower IC50 in the si-FOXD2-AS1 group compared to that in the control group. Transwell and wound-healing assays also showed suppressed invasion and migration by cisplatin-resistant OS cells when FOXD2-AS1 was knocked down. OS resistance to cisplatin was also inhibited by knocking down lncRNA NCK-AS1. Cheng Y et al. (2019) transfected shRNA-NCK1-AS1 into DDP-resistant MG63 cells to knock down lncRNA NCK-AS1. Cell growth, cell migration, and cell invasion were extremely inhibited in DDP-resistant MG63 cells after NCK-AS1 knockdown in the CCK-8, wound healing, and transwell assays, respectively. Cheng F-H et al. (2019) reported increased lncRNA ROR expression in cisplatin-resistant OS cells and transfected shRNA targeting ROR into cisplatin-resistant OS cells (MG63/DDP and U2OS/DDP). The MTT and transwell assays showed reduced cell proliferation and invasion in MG63/DDP and U2OS/DDP cells following ROR knockdown. This indicated that ROR knockdown suppressed cisplatin resistance in OS cells. Wen et al. (2020) observed decreased lncRNA SARCC expression in OS cells and transfected lncRNA SARCC into SaoS-2 and U2OS cells. When SaoS-2 and U2OS cells were exposed to cisplatin, cells overexpressing SARCC showed more impaired cell viability compared to cells without SARCC overexpression. They also demonstrated that OS cells sensitized to cisplatin with miR-143 overexpression and lncRNA SARCC upregulated miR-143.

### 4.2 LncRNAs as potential treatment targets to suppress doxorubicin resistance in OS

LncRNAs may also be treatment targets for doxorubicin resistance in OS. Gu Z et al. (2020) reported that lncRNA LINC00922 accelerated OS doxorubicin (DXR) resistance *via* the TFAP2C/LINC00922/miR-424-5p feedback loop. When they transfected short hairpin RNA (shRNA) specifically targeting LINC00922 into DXR-resistant OS cells (MG63/DXR), the CCK-8 assay showed inhibition of the IC 50 (50% inhibitory concentration). *In vivo*, a mouse xenograft model showed that tumor volume and weight were also inhibited by LINC00922 knockdown in MG63/DXR cells. Shen and Cheng (2020) reported that lncRNA ARSR enhanced OS adriamycin resistance by upregulating multidrug resistance-associated protein-1 (MRP1) through AKT activation. After transfected U2OS and MG63 cells with siRNA targeting the lncRNA ARSR, the cells were exposed to adriamycin at stepwise increasing concentrations. Cells expressing siRNA targeting lncRNA ARSR showed inhibited cell proliferation, an inhibitory effect that was enhanced with increased adriamycin concentrations. In their ADM-resistant OS mouse model, the suppression of tumor growth by ADM was recovered by si-lncARSR, and tumor growth was significantly suppressed in the si-lncARSR group compared to the siRNA-negative control group. Zhu et al. (2020) focused on the relationship between lncRNA Sox2OT-V7 and doxorubicin (DOX) resistance in OS and transfected a Sox2OT-V7-silenced lentivirus recombinant plasmid into U2OS cells. They reported an IC50 value of 1.533 µM in DOX-resistant U2OS (U2OS/DOX) cells after Sox2OT-V7 silenced plasmid transfection, compared to 4.380 µM in non-transfected DOX-resistant U2OS cells. Finally, they suggested that Sox2OT-V7 silencing sensitized U2OS/DOX cells *via* upregulated expression of miR-142/miR-22 and regulated autophagy in U2OS cells. Hu et al. (2021) reported that lncRNA ANCR exosomes from doxorubicin (DOX)-resistant U2OS cells induced drug resistance among Dox-sensitive cells. When the authors transfected siRNA targeting lncRNA ANCR into DOX-resistant KHOS and U2OS cells, MTT assays showed a significantly lower IC50 value in the ANCR-silenced group compared to that in the control group. This indicated that ANCR silencing could sensitize OS to DOX.

### 4.3 A single lncRNA can reduce multiple chemotherapy resistances

Some lncRNAs play a vital role in multidrug resistance. Pu et al. (2021) reported that the lncRNA LAMTOR5-AS1 is upregulated in chemotherapy-sensitive cells and transfected LAMTOR5-AS1 into OS cells. After exposure to IC 50 doses of VP-16 (etoposide), CBP (carboplatin), and DDP (cisplatin), the CCK-8 assay showed increased cell death in the LAMTOR5-AS1 group compared to the negative control. An annexin V-FITC staining assay showed that LAMTOR5-AS1 overexpression promoted DDP-induced apoptosis of SJSA-1 cells. *In vivo*, a tumor xenograft mouse model was constructed, in which LAMTOR5-AS1 overexpression promoted the inhibitory effects of DDP on tumor formation, weight, and volume. Sun et al. (2020) discovered that OIP5-AS1 served as a sponge of miR-137-3p and that OIP5-AS1 suppression reduced OS resistance to doxorubicin. They transfected siRNAs targeting OIP5-AS1 into DOX-resistant MG63 cells. The CCK-8 assay and flow cytometry analysis showed that OIP5-AS1 inhibition not only sensitized DOX-resistant MG63 (MG63/DOX) cells to doxorubicin-mediated cytotoxicity but also promoted the apoptosis of MG63/DOX cells. *In vivo*, compared to the control group, MG63/DOX cells transfected with lenti-sh-OIP5-AS1 grew slower and had smaller tumor volumes. LncRNA OIP5-AS1 also exhibited the ability to reduce cisplatin resistance in OS cells. Liu and Wang (2020) transfected siRNA against OIP5-AS1 into MG63 and Saos-2 cells and proved that OIP5-AS1 knockdown could enhance cisplatin sensitivity in OS cells *in vitro* and *in vivo*.

## 5 Discussion

OS is one of the most common malignant tumors in orthopedics, is highly aggressive, and is always transferred early. However, existing treatments for OS have many shortcomings, such as wide surgical resection ranges, a high risk of postoperative recurrence, and chemotherapy resistance. Recent research has revealed the potentially vital role of lncRNAs in OS progression. This review broadly divided lncRNAs into two categories: oncogenic lncRNAs and tumor-suppressive lncRNAs. Oncogenic lncRNAs are highly expressed in OS cells, while the expression levels of tumor-suppressive lncRNAs are low (Fei et al., 2019; Li et al., 2019; Wan et al., 2020; Zhou et al., 2020). After oncogenic lncRNA knockdown, several aspects of OS progression can be inhibited, including inhibited proliferation, arrested cell cycle at G0/G1 phase, reduced angiogenesis, suppressed invasion, and weakened migration ability. These anti-osteosarcoma effects of lncRNA were not only confirmed *in vitro* but have also been demonstrated *in vivo*. However, inhibiting OS progression required enhanced expression of tumor-suppressive lncRNAs (Chen L. et al., 2020; Huang et al., 2022). Moreover, regardless of oncogenic or tumor-suppressive lncRNAs, most of their mechanisms of action are to act as ceRNAs to miRNAs and then affect the expression of downstream proteins (Figure 1).

Although chemotherapy is an important tool in the treatment of OS today, especially in combination with surgery, which can narrow the scope of surgery and reduce the recurrence rate, chemotherapy resistance is still an inevitable topic. To find a new method to reduce the chemotherapy resistance of OS, researchers have turned to lncRNAs, with rich results. LncRNA FOXD2-AS1, NCK-AS1, and ROR were highly expressed in cisplatin-resistant OS cells, and cisplatin resistance in OS cells improved after their silencing (Cheng Y et al., 2019; Cheng F-H et al., 2019; Zhang Q-Q et al., 2021). Similarly, silencing LINC00922, lncARSR, Sox2OT-V7, and ANCR could improve doxorubicin resistance in OS. Moreover, silencing lncRNA LAMTOR5-AS1 and lncRNA OIP5-AS1 may also promote multiple kinds of chemotherapy resistance.

Although studies on osteosarcoma-related lncRNAs have achieved fruitful results, there remain many deficiencies in lncRNAs as targets for the treatment of osteosarcoma. First, the current research on osteosarcoma-related lncRNAs is still in the experimental stage, and clinical evidence for the treatment of osteosarcoma by lncRNAs is lacking. Second, few studies have assessed targeted delivery methods of lncRNAs or their silencers in osteosarcoma. Huang et al. (2022) purified and collected exosomes from osteosarcoma cells transfected with lncRNA MEG3. These exosomes were rich in lncRNA MEG3 and were modified with cRGD peptides to enhance their targeting capability. After the xenograft osteosarcoma model was established, fluorescently labeled exosomes were intravenously injected. The results showed that compared to the MEG3 group, the cRGD-modified exosomes emitted stronger fluorescence signals in the tumor site, confirming that the cRGD-modified exosomes had a stronger ability to target lncRNA delivery to osteosarcoma. However, most studies established animal models of osteosarcoma suppression using osteosarcoma cells transfected with lncRNA or lncRNA silencer plasmids rather than the targeted delivery of lncRNAs and their silencer. Third, while many lncRNAs are potential therapeutic targets for osteosarcoma, there remains a lack of standardized evaluation of efficacy and transverse comparison of efficacy.

The future research directions in osteosarcoma-related lncRNAs include more effective methods of targeted delivery of lncRNAs to osteosarcoma. Second, evaluation criteria for the effectiveness of targeted lncRNAs in the treatment of osteosarcoma are needed. Third, whether for diagnosis, treatment, or prognosis, we hope to see the clinical application of lncRNAs in osteosarcoma.
